# Re-Emergence of a West Nile Virus (WNV) Variant in South Spain with Rapid Spread Capacity

**DOI:** 10.3390/v15122372

**Published:** 2023-12-01

**Authors:** María José Ruiz-López, Pilar Aguilera-Sepúlveda, Sonia Cebrián-Camisón, Jordi Figuerola, Sergio Magallanes, Sarai Varona, Isabel Cuesta, Cristina Cano-Gómez, Patricia Sánchez-Mora, Juan Camacho, Carolina Sánchez-Peña, Francisco José Marchena, Ulises Ameyugo, Santiago Ruíz, María Paz Sánchez-Seco, Montserrat Agüero, Miguel Ángel Jiménez-Clavero, Jovita Fernández-Pinero, Ana Vázquez

**Affiliations:** 1 Estación Biológica de Doñana—CSIC, Avda. Américo Vespucio 26, 41092 Sevilla, Spain; sonia.cebrian@ebd.csic.es (S.C.-C.); sergio.magallanes@ebd.csic.es (S.M.); 2 CIBER de Epidemiología y Salud Pública (CIBERESP), 28029 Madrid, Spain; 3 Centro de Investigación en Sanidad Animal (CISA-INIA), CSIC, 28130 Valdeolmos, Spain; aguilera.pilar@inia.csic.es (P.A.-S.);; 4 Unidad Bioinformática, Unidades Centrales Científico-Técnicas, Instituto de Salud Carlos III, 28220 Madrid, Spain; s.varona@isciii.es (S.V.); isabel.cuesta@isciii.es (I.C.); 5 Escuela Internacional de Doctorado de la UNED (EIDUNED), Universidad Nacional de Educación a Distancia (UNED), 28232 Madrid, Spain; 6 Laboratorio Central de Veterinaria (LCV), Ministry of Agriculture, Fisheries and Food, Algete, 28110 Madrid, Spain; 7 Centro Nacional de Microbiología, Instituto de Salud Carlos III, CNM-ISCIII, Carretera Pozuelo-Majadahonda, Km. 2.2, Majadahonda, 28220 Madrid, Spain; patrisanchezm98@gmail.com (P.S.-M.);; 8 CIBER de Enfermedades Infecciosas (CIBERINFEC), 28029 Madrid, Spain; 9 Junta de Andalucía, Consejería de Salud y Familias, Dirección General de Salud Pública y Ordenación Farmaceútica, Subdirección de Protección de la Salud, 41020 Sevilla, Spain; 10 Servicio de Control de Mosquitos de la Diputación Provincial de Huelva, Ctra. Hospital Infanta Elena s/n, 21007 Huelva, Spain

**Keywords:** *Culex*, flavivirus, genetic variants, Spain, vector-borne diseases, WNV lineage 1, zoonosis

## Abstract

West Nile Virus (WNV) is a mosquito vector-borne zoonosis with an increasing incidence in Europe that has become a public health concern. In Spain, although local circulation has been known for decades, until 2020, when a large outbreak occurred, West Nile Virus cases were scarce and mostly occurred in southern Spain. Since then, there have been new cases every year and the pathogen has spread to new regions. Thus, monitoring of circulating variants and lineages plays a fundamental role in understanding WNV evolution, spread and dynamics. In this study, we sequenced WNV consensus genomes from mosquito pools captured in 2022 as part of a newly implemented surveillance program in southern Spain and compared it to other European, African and Spanish sequences. Characterization of WNV genomes in mosquitoes captured in 2022 reveals the co-circulation of two WNV lineage 1 variants, the one that caused the outbreak in 2020 and another variant that is closely related to variants reported in Spain in 2012, France in 2015, Italy in 2021–2022 and Senegal in 2012–2018. The geographic distribution of these variants indicates that WNV L1 dynamics in southern Europe include an alternating dominance of variants in some territories.

## 1. Introduction

The presence of West Nile Virus (WNV) in Europe has been known since 1962 [1]. In Spain, WNV circulation was confirmed in the early 2000s [2,3,4,5,6]. Despite the confirmed circulation, cases in humans were rare until 2020 and occurred only in southwestern Spain (one case in 2004, two in 2010 and three in 2016). In the summer of 2020, the largest WNV outbreak in Spain occurred in this area, with 77 symptomatic reported cases in humans and eight deaths [7,8]. The outbreak predominantly affected localities in the province of Seville, although there were also isolated human cases in nearby provinces, such as Cádiz. In 2021, the six human cases reported occurred in southwestern Spain (province of Seville) [9]. In 2022, two of the four reported cases occurred in this area as well (provinces of Cádiz and Córdoba) and the other two occurred in the northeast of Spain [10].

So far, three different West Nile Virus lineages have been detected in Spain. In 2006, putative lineage 6 was detected in mosquitoes from Andalusia [5]. Since 2008, different variants of WNV lineage 1 (L1) have been repeatedly identified in mosquitoes, horses and birds, in southwestern Spain. The human cases in the 2020 outbreak, as well as other WNV foci affecting animals in nearby areas, were caused by lineage 1 (L1) strains [11]. Finally, more recently, in northeastern Spain, far away from these L1 foci, WNV lineage 2 (L2) has been detected circulating at least since 2017 [12,13], causing outbreaks in animals. Because no other WNV lineage has been detected in the region, it is thought that the human cases that occurred in the area in 2022 are caused by L2, but further information is needed [14]. Previous analyses of WNV L1 strains from Spain [15] provided sound evidence that the virus may remain endemic in the area for years, rather than being introduced periodically (e.g., through migratory birds). A single monophyletic clade was extended amongst all western Mediterranean territories, giving rise to a range of genetic variants moving around the area [13]. In this scenario, access to complete genomes of the WNV strains that are circulating in endemic areas is crucial to better understanding WNV geographical spread and amplification. Virus surveillance in mosquitoes constitutes a useful tool for characterization of the genomes, enabling accurate determination of the lineages and variants that are circulating in each specific area even in the absence of reported human or animal cases. 

Here, we characterized four genome sequences from WNV-positive samples from mosquitoes collected in 2022 in the provinces of Seville and Cádiz. We focused on these areas because in both provinces, there were human cases in 2020, and there were cases either in 2021 (Seville) or 2022 (Cádiz) [10]. In addition, we also obtained the whole genome sequences of two archived WNV isolates from a horse affected in an outbreak that occurred in 2012 also in Cádiz. Thus, for this area, we have WNV genomic information for several years that comes both from human cases, horses and mosquito surveillance. We compared these five full genomes with other publicly available genomes representative of different L1 clusters, specifically incorporating all existing sequences from Spain, and re-presentative sequences from other western Mediterranean countries and Africa, either from horses, birds, mosquitoes or human samples. 

## 2. Materials and Methods

### 2.1. Analysis of WNV in Mosquitoes

**Figure 1:**
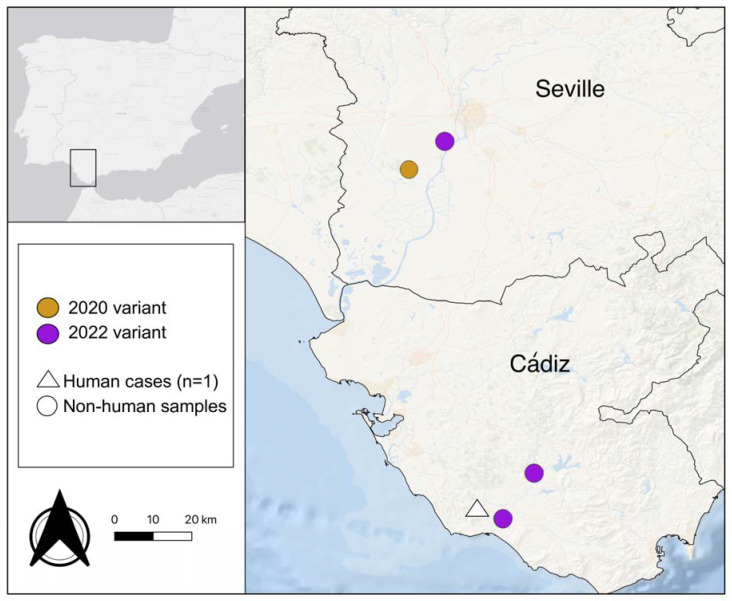
Map of the provinces of Cádiz and Seville showing the sampling locations for the 4 mosquito pools sequenced to obtained WNV genomes (circles). The colors represent the genomic variants identified. The location of the reported human case that occurred in Cádiz in 2022 is also shown (triangle).

We analyzed mosquitoes captured during the summer and fall of 2022 in 20 localities of the provinces of Cádiz and Seville (Andalusia, Spain). In each locality, we placed one BG-Sentinel trap (BioGents AG, Regensburg, Bayern, Germany), which is a collapsible fabric container that has a lid with a hole in the middle. The trap has an electrical fan that sucks air into the trap through the lid hole and a pipe, catching the approaching mosquitoes into a bag. The BG traps operated for 24 h each week and were baited with approximately 1 kg of dry ice to generate a continuous flow of CO_2_ at the entry of the trap and lure the mosquitoes. Captured mosquitoes were transported in dry ice to the laboratory and stored at −80 °C until further processing. Species identification was carried out using MosKeyTool [16], and mosquitoes were pooled in groups of up to 50 females by species, date and locality. Mosquito pools were processed to extract RNA using a Maxwell^®^ extraction robot and the Viral Total Nucleic Acid Purification kit (Promega, Madison, WI, USA). WNV presence was determined with qRT-PCR for 3102 mosquito pools following Vázquez et al. [17]. After a preliminary screening with the NS5 gene [6], we selected four positive samples (JA22 423 and JA22 544 from Cádiz and 22C2285 and 22C2392 from Seville; Figure 1) that had a Ct lower than 30 to carry out genome sequencing. JA22423 and JA22544 genomes were sequenced directly from the mosquito pool. For 22C285 and 22C392, we carried out viral isolation on Vero E6 cells. For the whole genome sequencing, the library was prepared using the NEBNext^®^ Ultra II RNA Library Prep Kit for Illumina^®^ with NEBNext^®^ Multiplex Oligos for Illumina^®^ Index Primers Set 3 (New England BioLabs Inc., USA) and sequenced directly on an Illumina MiSeq v2 (300 cycles). Sequences were analyzed for viral consensus genome reconstruction using the pipeline described by Ruiz-López et al. [18].

### 2.2. Analysis of WNV in the Horse

Horse samples were collected from the brain and cerebrospinal fluid of an infected horse from Cádiz, during an outbreak in the 2012 transmission season. Because West Nile Virus occurs as a population of genetic variants (quasispecies) [19], we processed the samples of both tissues to study the level of microvariation. These samples were further homogenized and subjected to virus isolation as described in [2]. Thus, two isolates were obtained: SPA12-01_Spain/2012/H-b/2V and SPA12-02_Spain/2012/H-cf/2V (from now on, SPA12-01 and SPA12-02). These archived isolates were fully sequenced using a Sanger sequencing approach as previously described by [15].

### 2.3. Nucleotide Sequencing and Phylogenetic Analyses

We compared the genomes with all WNV L1 genomes from Spain available at GenBank (n = 11) as well as with representative sequences from different genetic L1 clusters (n = 67). Multiple alignments were carried out with Clustal W and the phylogenetic tree was generated using a maximum likelihood approach and a general time reversible model (GTR+G+I) with 1000 bootstraps in MEGA11 (Molecular Evolutionary Genetics Analysis version 11) [20].

## 3. Results

**Figure 2:**
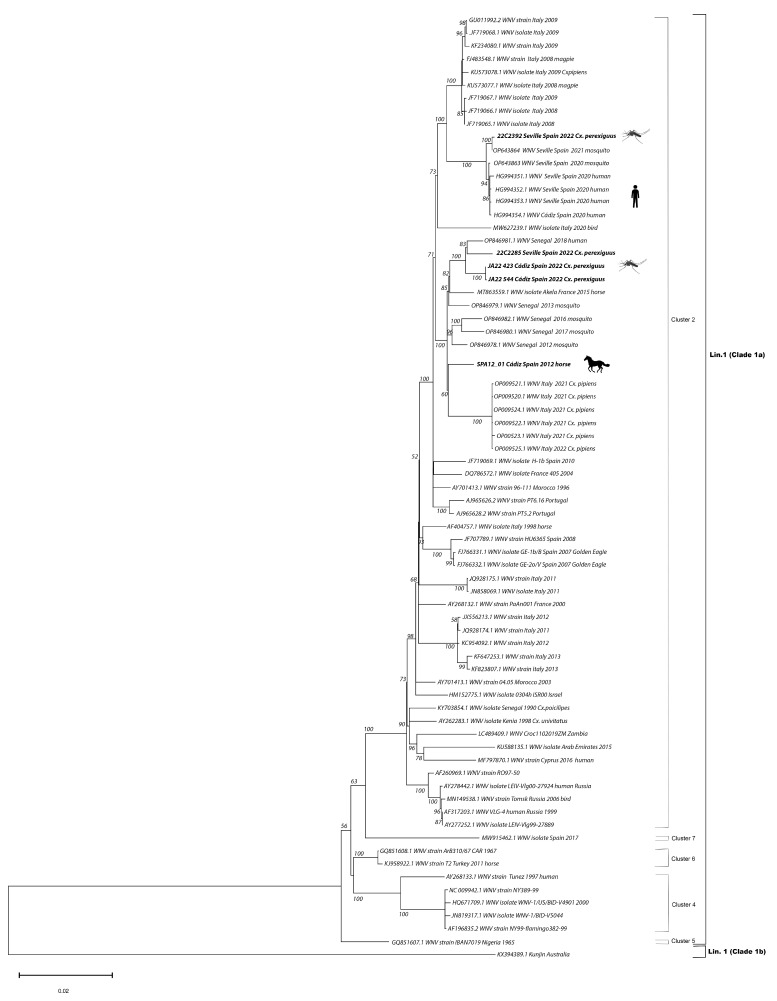
Phylogenetic analyses of 72 complete genomes of West Nile Virus (WNV). Bootstrap values over 50 are given for 1000 replicates. Viral sequences are identified by GenBank accession number. Sequences in bold were generated in this study.

The two isolates obtained from the horse sick in 2012 (SPA12-01: GenBank Accession # OM302323 and SPA12-02: GenBank Accession # OM302322) had a percentage of nucleotide identity for the complete sequence > 99.999%. Thus, we ran the maximum likelihood analyses with only one of the sequences. Maximum likelihood phylogenetic analyses confirmed that all the full genome sequences obtained in this study belong to WNV lineage 1, clade 1a, cluster 2, Mediterranean subtype [21,22] (Figure 2). The two sequenced genomes obtained from the two mosquito pools collected in Cádiz in 2022, JA22 423 (GenBank Accession # OQ357821) and JA22_544 (GenBank Accession # OQ357822), grouped with the isolate obtained from the horse sample collected in Cádiz in 2012 (SPA12-01: GenBank Accession # OM302323). Additionally, they grouped with a French isolate from 2015 [23], with the 2021 and 2022 Italian genomes that have been associated with a recent large outbreak in Italy [24] and with several sequences from Senegal, obtained both from human and mosquitos between 2012 and 2018 [25]. In all cases, the percentage nucleotide identity for the complete sequences was >98%. In contrast, out of the two 2022 full genomes sequenced from Seville, one of them (22C2285: GenBank Accession # OQ357820) grouped with the 2012–2022 Cádiz genomes, while the other one (22C2392: GenBank Accession # OQ357819) grouped with the sequences detected in 2020 and 2021 from human and mosquito samples [18].

## 4. Discussion

The results of this study reveal a re-occurrence of a variant 10 years after its first detection (2012) in the same area (Cádiz). Strains highly related to this variant have been reported previously in France (2015) [23], Senegal (2012–2018) [25] and later Italy (2021–2022) [24], showing a remarkable spread capacity. Thus, two possible scenarios may explain these observations: (i) this variant might have been circulating silently since 2012 or (ii) it might have re-entered Spain from other regions in either Europe or Africa, possibly through bird movements. The pathogenic potential of this variant became evident from its first appearance, i.e., in the two samples collected from a sick horse during an equine outbreak in Cádiz in 2012. Indeed, the closely related sequence identified in France was obtained from a horse suffering from West Nile neuro-invasive disease during an outbreak in the Camargue area in 2015 [23]. In Senegal, where this variant has been circulating in mosquitoes, a human case was also detected in 2018 [18]. Finally, this variant has also been detected in Italy in 2021 and 2022, years in which an important outbreak occurred with 723 reported human cases of West Nile neuroinvasive disease [10]. This outbreak has also been linked to the presence of L1 [24,26]. In addition, our results also confirm that this variant has spread from Cádiz to Seville, where it had never been detected before, and it is now co-circulating with the variant that caused the 2020 human outbreak.

Overall, this study suggests that in Cádiz, a rapid shift has occurred from the WNV variant identified during the large outbreak in 2020, known to have caused human cases [12], and the clearly distinguishable variant circulating in 2022, which could be dominating the next transmission seasons in this area and that is expanding to other areas. Meanwhile, in Seville, there was co-circulation of both variants in the 2022 season. In light of these findings, we hypothesize that multiple WNV variants can co-circulate in the same territory either as they re-emerge over years after silent circulation or as they are re-introduced from other territories. In this way, alternation of strains dominating transmission may occur, leading to episodic outbreaks in horses or humans triggered by specific environmental conditions still not well characterized.

## 5. Conclusions

The integration of vector and viral surveillance is key to provide early warning signals to inform vector control programs and reduce WNV impact. Here, mosquito surveillance has allowed the characterization of the variants of WNV circulating in an endemic area and has revealed the re-emergence of a variant that was previously detected in 2012 in the same region of southern Spain. These results underscore the usefulness of effective surveillance programs such as the one implemented by the health authority of Andalusia that includes sequential monitoring of mosquitoes, where WNV was detected well in advance to the first human cases [7,27]. In addition, the results show the usefulness of monitoring WNV sequence variants, for which mosquitoes represent a reliable source in areas of active virus circulation. To fully understand the ecological and evolutionary history of these variants, we need to increase the number of WNV sequenced genomes both at local, national and supranational levels and encompassing multiple years to obtain a clear picture of WNV dynamics in Europe and Africa.

## Data Availability

The data presented in this study are openly available in GENBANK.
